# Post-pandemic resurgence of Mycoplasma pneumoniae and its emerging link with Rhinovirus in children

**DOI:** 10.1007/s15010-026-02833-8

**Published:** 2026-05-27

**Authors:** Eva Brandlmeier, Cora Nagl, Jimmy Omony, Laura Kolberg, Melanie Meyer-Bühn, Constanze Jakwerth, Bianca Schaub, Ulrich von Both, Nicole Maison

**Affiliations:** 1 Center of Allergy and Environment (ZAUM), School of Medicine and Health, Technical University of Munich (TUM) and Helmholtz Munich, Member of the German Center for Lung Research (DZL), Munich, Germany; 2 Department of Pulmonary and Allergy, Dept. of Pediatrics, Dr von Hauner Children’s Hospital, University Children´s Hospital, Ludwig-Maximilians- University, Munich, Germany; 3 Department of Infectious Diseases, Dr. von Hauner Children’s Hospital, LMU University Hospital, Ludwig-Maximilians-Universität München (LMU), Munich, Germany; 4 Comprehensive Pneumology Center (CPC-M), University of Munich, LMU Munich, German Center for Lung Research (DZL), Munich, Germany; 5 German Center for Infection Research (DZIF), Partner Site Munich, Munich, Germany; 6 Institute of Asthma and Allergy Prevention (IAP), Helmholtz Munich – German Research Center for Environmental Health (GmbH), Munich, Germany; 7 German Center for Child and Adolescent Health (DZKJ), Dr von Hauner Children’s Hospital, LMU Munich, Munich, Germany

**Keywords:** Mycoplasma pneumonia, Rhinovirus, Infection, Pandemic, Epidemiology

## Abstract

**Background:**

Mycoplasma pneumoniae (MPP) infections occur regularly every 1 to 3 years. A wave of infections was anticipated around late 2022 to early 2023. However, our data revealed a delayed onset, with a notable increase in cases in 2023/24. This study describes pediatric MPP detections during this resurgence and explores clinical differences between MPP mono-detection and MPP with rhinovirus/enterovirus (RV/EV) co-detection.

**Methods:**

We retrospectively analyzed PCR-confirmed MPP detections in children aged 0–17 years presenting to a tertiary pediatric hospital between 2017 and 2024. Cases from 2017 to 2022 were used as descriptive historical background data (G1, *n* = 10), while the main analysis focused on the 2023/24 cohort (G2, *n* = 87). Within this cohort, children with MPP mono-detection were compared with those with MPP plus RV/EV co-detection. Group comparisons were performed using chi-square or Fisher’s exact test and t-test or Mann–Whitney U test, as appropriate.

**Results:**

Ten sporadic MPP detections occurred between 2017 and 2022, compared with 87 detections in 2023/24, indicating a marked increase in hospital presentations. Because of the small historical group, comparisons with 2023/24 were interpreted descriptively. In the 2023/24 cohort, RV/EV was the most frequent viral co-detection. Compared with children with MPP mono-detection, children with MPP plus RV/EV co-detection were more often male, were less frequently febrile, and showed lower inflammatory parameters, including lower rates of elevated CRP and higher rates of leukopenia and neutropenia.

**Conclusion:**

In 2023/24, Mycoplasma pneumoniae infections surged dramatically, accompanied by an unprecedented rise in Rhino/Enterovirus coinfections especially in young, male patients. While these findings highlight a potentially important phenotypic pattern, further prospective studies are needed to clarify causality, temporal sequence, and distinct underlying mechanisms.

**Supplementary Information:**

The online version contains supplementary material available at 10.1007/s15010-026-02833-8.

## Introduction

*Mycoplasma pneumoniae* (MPP) is one of the most common pathogens causing atypical pneumonia [1]. As a bacterium lacking a cell wall, MPP is an obligate parasite that relies entirely on host cells for its survival [2, 3]. Through its adhesion organelle, MPP establishes a strong attachment and association with the host cell [4]. Epithelial cells of the upper and lower respiratory tract serve as the main host sites for the pathogen [2]. Because of MPP’s unique characteristics, the immune response required to eliminate the infection is of particular importance [5].

Atypical pneumonia—also known as “walking pneumonia”—was first described in the 1930s [6]. The main distinction between typical pneumonia, mostly caused by *Streptococcus pneumoniae*, and atypical pneumonia is seen in the clinical presentation, which is generally milder [7, 8], and in the absence of characteristic findings such as lobar consolidation on chest X-ray [9]. Typical respiratory tract infections caused by MPP often begin with mild symptoms such as sore throat, nasal congestion, or otitis media. The progression to atypical pneumonia can be gradual and is characterized by fever and cough [10]. In adults, MPP infections may even remain asymptomatic in up to 20% of cases [11].

MPP infections occur most frequently in late childhood and early adulthood [12], although all age groups can be affected. Since transmission occurs via respiratory droplets, a correlation is assumed between close contact in community settings [13]. Globally, MPP infections show a cyclical pattern with interval durations reported to range from about one to seven years [14, 15]. In Germany, the most recent increase in reported MPP infections occurred in 2018 [16]. During the COVID-19 pandemic (2020–2023) [17], however, there was a dramatic global decline in MPP detections throughout all stages of the pandemic [18, 19]. A delayed but sharp rise in MPP cases has been reported at the turn of the year 2023/2024 [20].

Similarly, other common respiratory pathogens also showed a decline in detection rates during the COVID-19 pandemic, particularly in association with non-pharmaceutical interventions (NPIs). These included viral pathogens such as respiratory syncytial virus (RSV), influenza virus, and human metapneumovirus and bacterial pathogens such as *Streptococcus pneumoniae*,* Haemophilus influenzae*,* Bordetella pertussis*, and *Corynebacterium diphtheriae*. In contrast to MPP, detection rates for these bacterial pathogens increased rapidly following the easing of NPIs or in the later stages of the COVID-19 pandemic [21]. However, other pathogens like adenovirus and rhinovirus showed a constant or even an increase in detection levels even during periods of strict NPI implementation [22].

Building on this epidemiological context, we describe pediatric PCR-confirmed *Mycoplasma pneumoniae* detections at a tertiary children’s hospital during the 2023/24 resurgence. Cases from 2017 to 2022 were used as descriptive historical background because only few sporadic detections occurred during this period. The primary focus of the present analysis was the 2023/24 cohort, with particular emphasis on clinical and laboratory differences between children with MPP mono-detection and those with MPP plus RV/EV co-detection.

## Methods

A retrospective analysis was conducted of all direct pathogen detections positive for Mycoplasma *pneumoniae* obtained during routine clinical practice, in children 0–18 years of age, presenting at the hospital due to an acute infection, primarily of the upper and lower respiratory tract. Specimens included nasal swabs (*n* = 51), throat/tonsillar swabs (*n* = 21), unspecified swabs (*n* = 11), nasopharyngeal swabs (*n* = 7), sputum samples (*n* = 2), and bronchoalveolar lavage (BAL) specimens (*n* = 2), while the specimen type was not documented in three cases. Detection of respiratory pathogens was performed using multiplex PCR (BioFire^®^ FilmArray^®^ Respiratory Panel 2.1. bioMérieux. Salt Lake City. UT. USA) which detects, among others, adenovirus, seasonal coronaviruses (229E, HKU1, NL63, OC43), SARS-CoV-2, MERS-CoV, influenza A/B, parainfluenza 1–4, RSV, human metapneumovirus, R/EV, *Bordetella pertussis/parapertussis*,* Chlamydophila pneumoniae (C. pneumoniae)*, and MPP.

The BioFire^®^ FilmArray^®^ Pneumonia Panel (bioMérieux) was used for a separate analysis of pneumonia pathogens. The panel tests for typical and atypical pneumonia pathogens and selected respiratory viruses. The choice of panels was based on clinical decisions. Regardless of the panel applied, in the case of a positive detection of MPP, adenovirus, coronaviruses 229E, NL63, OC43, HKU1 (excluding SARS-CoV-2), human metapneumovirus, R/EV, influenza A (including subtypes), influenza B, parainfluenza viruses 1–4, RSV, and *C. pneumoniae* were determined in parallel.

Overall, 239 children with suspected MPP infections based on symptoms of a respiratory tract infection were seen in 2017–2024. Initially, 134 children were excluded due to either incomplete retrospective data, clinical diagnosis only, or detection methods other than PCR testing. Subjects older than 18 years (*n* = 4) and duplicate cases (*n* = 4) were excluded **(Figure S1)**. A duplicate was defined as a repeated detection of MPP in the same child within a period of less than one month.

For MPP-positive cases, the clinical course was retrospectively analyzed. Treatment-related factors—including reason for presentation, hospitalization, length of stay, and presenting symptoms—were assessed, and both diagnostic and therapeutic parameters were analyzed.

Laboratory values were standardized based on the timing of pathogen detection. For cases where MPP was detected within 3 days of admission, initial admission laboratory results were used. For all other cases, the laboratory value closest to the time of detection (within a window of -2 to 0 days) was analyzed. Not all patients underwent laboratory testing for reasons that could not be determined retrospectively (Laboratory testing G1: *n* = 10, G2: *n* = 77). One patient did not meet the standardization criteria and was excluded from analysis.

The main comparative analysis focused on the 2023/24 cohort and compared children with MPP mono-detection with those with MPP plus RV/EV co-detection. Given the retrospective design, modest subgroup sizes, and multiple exploratory comparisons, all subgroup findings were interpreted as hypothesis-generating. Missing values were excluded on a variable-by-variable basis, and denominators are reported where applicable.

The MPP^N^ group was defined as patients who only had MPP as a confirmed pathogen. Patients who had both MPP and, additionally, RV/EV alone were classified into the MPP^RV^ group. In two cases, patients had additional pathogens besides RV/EV codetection; these patients were excluded from the analysis to observe the effect of RV/EV alone.

Statistical analyses were performed using IBM SPSS Statistics, version 29.0 (IBM Corp. Armonk. NY. USA). To compare the recent resurge numbers to those from the previous periods, during which only sporadic cases of MPP were observed, the cohort was divided into two groups: Group 1 (G1, 2017–2022) and Group 2 (G2, 2023–2024). We defined G1 as “pre-epidemic background incidence” and G2 as the “epidemic surge period,” aiming to reflect the observed epidemiological breakpoint. Because only ten MPP-positive cases were identified between 2017 and 2022, this historical group was considered descriptive background rather than a robust comparator. Statistical comparisons between G1 and G2 were therefore interpreted cautiously and used primarily to contextualize the observed increase in detections during 2023/24.

Descriptive statistics were used to summarize demographic, clinical, and laboratory characteristics. Continuous variables were summarized as mean (standard deviation, SD) and median (interquartile range, IQR), as appropriate. Categorical variables were presented as absolute and relative frequencies (%). Group comparisons were conducted using the Chi-square test or Fisher´s exact test (FET) for categorical variables, and the independent-samples t-test or Mann-Whitney U test (U-test) for continuous variables, depending on data distribution. A p-value < 0.05 was considered statistically significant. Multiple-response analyses were applied to variables allowing more than one response per patient (e.g., pre-existing conditions, symptoms, co-detections). Missing values were excluded from each analysis on a case-by-case basis.

To visualize co-detection patterns, Sankey diagrams were generated to depict the distribution of additional pathogen detections among children with MPP infection.

While each child was counted only once in the initial “co-detection” node, subsequent branches represent all detected pathogens per child. Consequently, multiple detections in the same patient are reflected as multiple flows, and the total number of flows may exceed the number of children with co-detections.

**Fig. 1:**
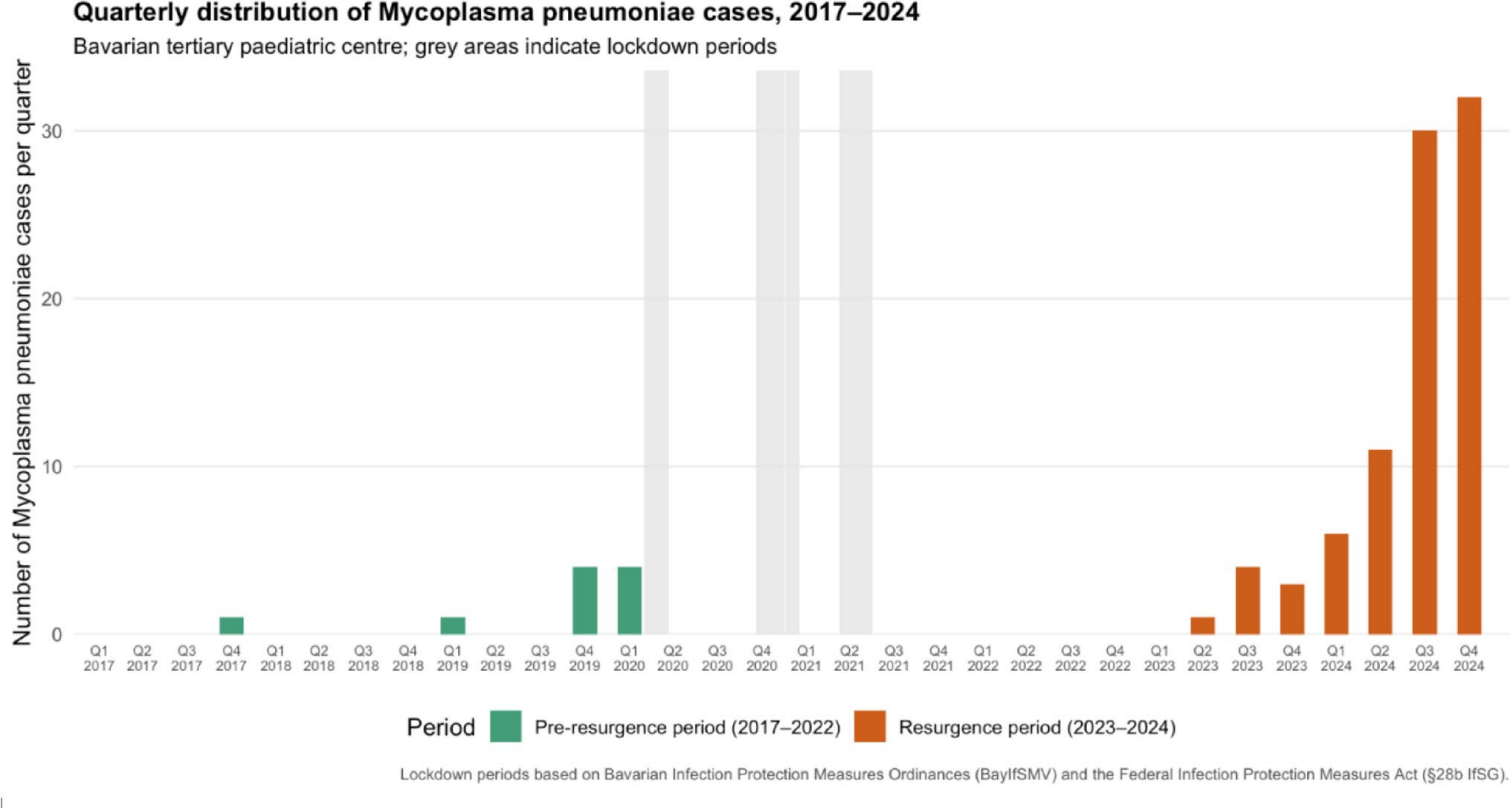
Quarterly distribution of MPP cases from 2017–2024; Before the first lockdown (indicated in grey) only sporadic cases presented at the Hospital with a maximum of *n* = 4/Q (green). In 2023 a rising number was observed with a maximum of *n* = 32/Q in Q4-2024

Furthermore, the graphical visualizations **(**Figs. 1 and 3**)** were created using R (version 4.5.2) [23] with the packages *ggplot2* [24], *dplyr* [25], and *tibble* [26].

## Results

### Epidemiological characteristics

A total of 97 children with PCR-confirmed MPP detection were included. Ten cases occurred between 2017 and 2022, whereas 87 cases were identified in 2023/24. This marked increase indicates a substantial rise in MPP-associated hospital presentations during the post-pandemic resurgence period. Because the historical group was small, comparisons between 2017 and 2022 and 2023/24 were interpreted descriptively and not considered sufficient to establish robust differences in clinical phenotype. While in 2017–2022 only sporadic cases were recorded (*n* = 10), there was a dramatic increase in hospital presentations, with 87 patients presenting in 2023/24 (Fig. 1). With a constant hospitalization rate (G1 70.0% vs. G2 75.9%; FET, *p* > 0.705), this led to a high number of hospitalized patients (*n* = 0,16/month in G1 vs. *n* = 2,75/month in G2). The mean duration of hospitalization showed high variability, particularly in G2 due to outliers (G1: one outlier of 51 days; G2: seven outliers, with a maximum duration of 158 days), whereas the median hospital stay was not significantly different between groups (7.0 days (IQR 12.0) G1 vs. 6.0 days (IQR 6.5) G2; U = 173.50, *p* = 0.302). Intensive care treatment was required in 20% in G1 and 6.3% in G2, respectively (FFH, *p* = 0.318). There was no significant difference in sex and age. Comorbidities were common in both groups, even though in G2 patients tended to be less affected by preexisting conditions (G1 80.0% vs. G2 57.1%, *p* = 0.193) (Table E2).

### Clinical pattern, diagnostics and treatment in MPP infections

In both groups, almost all patients presented with a focus on respiratory symptoms (G1 100% vs. G2 90.8%; FET *p* = 0.99), with cough being the most frequent symptom (100% vs. 83.9%; FET, *p* = 0.349). While wheezing occurred in 40% of all patients in G1, these symptoms were noted only in 23% in G2 (FET, *p* = 0.258). Gastrointestinal symptoms were reported in about half of all cases (50.0% vs. 49.4%; χ^2^ [1] = 0.002, *p* = 0.961), with vomiting being the most common. Almost all patients received antibiotics mainly targeted to MPP infections. In G1 patients more often received inhaled steroids (2 [20.0%] vs. 6 [6.9%]; FFH, *p* = 0.070) and less systemic steroids (1 [10.0%] vs. 21 [24.1%]; FFH, *p* = 0.171) (Table 1).

**Table 1:** . Patients’ clinical features compared between Group 1 (2017 – 2022) and Group 2 (2023 – 2024)

Constituents	Group 1 (G1, 2017-2022) *n* = 10	Group 2 (G2, 2023-2024) *n* = 87	*p*-value
Age (y) – median (IQR)	6.69 (5.50)	8.97 (7.98) (86)	0.260^U^
Female Gender	6 (60.0)	44 (51.2) (86)	0.743^FET^
Hospitalization	7 (70.0)	66 (75.9)	0.705^FET^
Duration of Hospitalization (d) – median (IQR)	7.00 (12.00)	6.00 (6.50)	0.302^U^
ICU Treatment	2 (20.0)	7 (8.9)	0.318^FFH^
Respiratory Infection as Main Complaint	5 (50.0)	59 (67.8)	0.301^FET^
**Symptoms**			
General Respiratory Symptoms	10 (100.0)	79 (90.8)	>0.999^FFH^
Cough	10 (100.0)	73 (83.9)	0.349^FET^
Wheeze	4 (40.0)	20 (23.0)	0.258^FET^
General Gastrointestinal Symptoms	5 (50.0)	43 (49.4)	>0.999^FET^
Diarrhea	2 (20.0)	11 (12.6)	0.619^FET^
Emesis	5 (50.0)	27 (31.0)	0.290^FET^
Fever > 38.5 °C	7 (70.0)	61 (70.1)	>0.999^FET^
**Pre-existing Conditions**	8 (80.0)	49 (57.1) (86)	0.193^FET^
Diagnostics			
Laboratory	10 (100.0)	77 (88.5)	0.592^FET^
Chest X-Ray	9 (90.0)	53 (60.9)	0.089^FET^
**Treatment**			
O2-Substitution	5 (50.0)	38 (43.7)	0.747^FET^
Inhaled NaCl	7 (70.0)	51 (58.6)	0.167^FFH^
Inhaled Salbutamol	5 (50.0)	28 (32.2)	0.374^FFH^
Inhaled Adrenalin	1 (10.0)	2 (2.3)	0.083^FFH^
Inhaled Atrovent	1 (10.0)	11 (12.6)	0.284^FFH^
Inhaled Corticosteroids	2 (20.0)	6 (6.9)	0.070^FFH^
Systemic Corticosteroids	1 (10.0)	21 (24.1)	0.171^FFH^
Antibiotics i.v./p.o.	10 (100.0)	85 (97.7)	>0.999^FET^

Values are presented as n (%) unless otherwise specified. Continuous variables are shown as median (IQR).p-values were calculated using the Fisher’s exact test (FET), Fisher–Freeman–Halton test (FFH), or Mann–Whitney U test (U), as appropriate

Because not all data could be reconstructed retrospectively, analyses were performed using a pairwise exclusion approach

Almost all patients in G1 received x-ray imaging, whereas only 60% were radiographically examined in G2 (9 [90.0%] vs. 53 [60.9%]; FET *p* = 0.089).

The most frequent laboratory abnormalities included elevated C-reactive protein (CrP) levels (8 [80%] vs. 51 [67.1%]; *p* = 0.495), decreased platelet counts (1 [11.1%] vs. 19 [25.3%]; FET, *p* = 0.679), and decreased eosinophil counts (4 [44.4%] vs. 28 [39.4%]; FET, *p* = 0.99), none of which reached statistical significance between G1 and G2 (Table E3).

### Co-detections in MPP patients

During MPP infection, approximately half of the patients tested positive for additional pathogens (G1 5 [50.0%] vs. G2 41 [47.1%]; FET, *p* > 0.999).

**Fig. 2:**
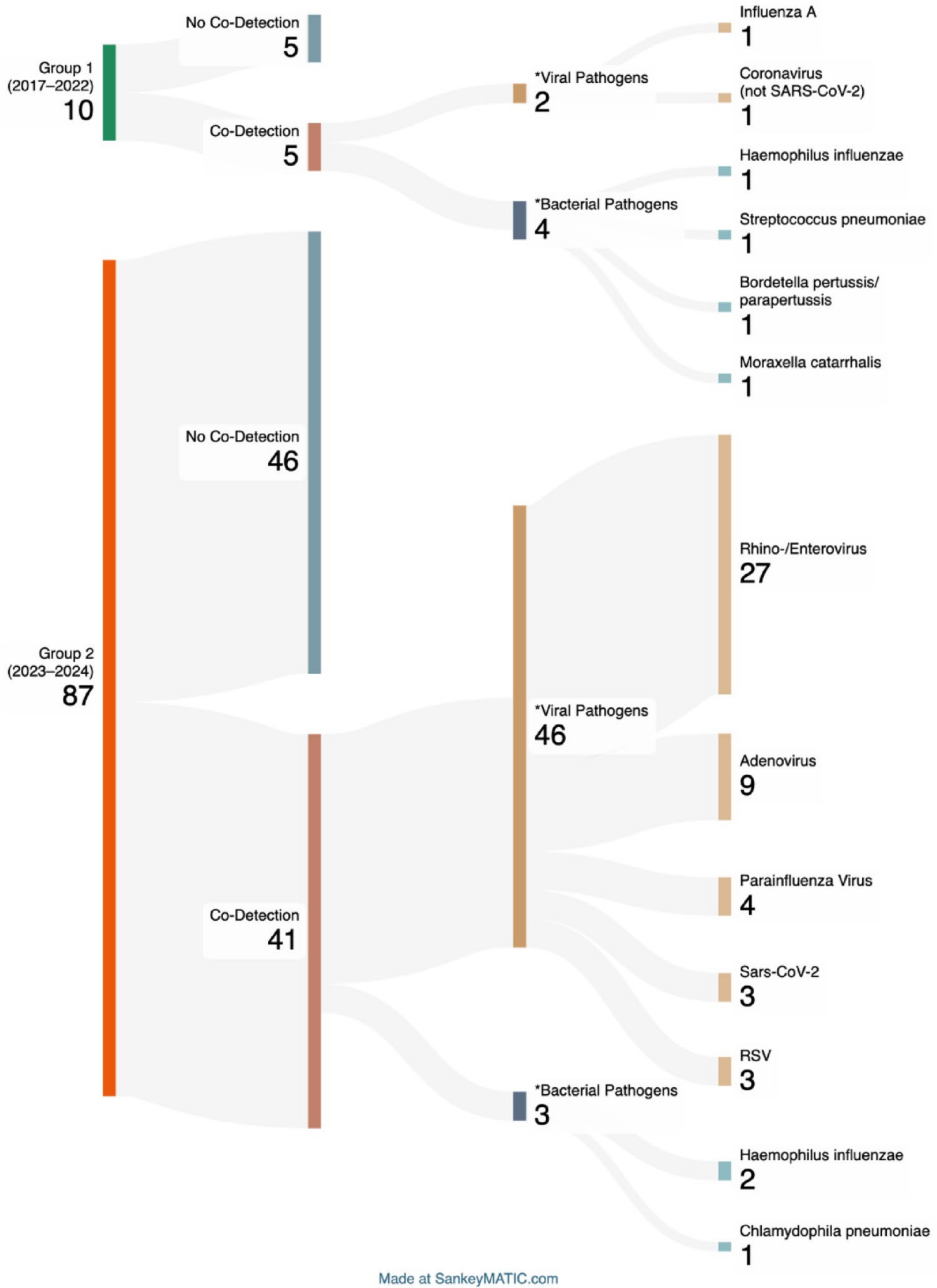
**A** Distribution of co-detected pathogens in children from 2017–2022**.** Of all pathogen detections, 67% were bacterial (4/6) and 33% viral (2/6).**B** Distribution of co-pathogens from 2023–2024. Of all pathogen detections, 94% were viral (46/49), primarily R/EV. Multiple co-infections per child were possible; therefore, the number of pathogen detections exceeds the number of children with co-infections

Most patients had a single co-detection (4/10 [40.0%] vs. 34/87 [39.1]), while only a few presented with more than one additional pathogen besides *Mycoplasma pneumoniae*. (Fig. 2**)**.

Between 2017 and 2022, bacterial co-detection was significantly more frequent compared to 2023/24 (3 [30.0%] vs. 2 [2.3%]; FET, *p* = 0.007). In contrast, viral co-detections were more common in 2023/24, although the difference was not statistically significant (2 [20.0%] vs. 39 [44.8%]; FET, *p* = 0.183). Additional mixed viral–bacterial co-detections occurred exclusively in G2 (1 [%]; FET, *p* > 0.99).

The spectrum of detected pathogens differed between the groups. Certain pathogens were found only in one group. Among pathogens present in both groups, detection rates varied (Table E4).

Of note, in 2017–2022 R/EV was not detected at all, in contrast, 27 (31%) patients tested positive in 2023/24 (*p* = 0.057) **(**Fig. 2**).**

### Clinical pattern of patients with MPP mono-infection (MPP^N^) versus MPP plus RV/EV Co-detection (MPP^RV^)

Due to the high co-detection rate with R/EV, a further analysis of potential differences between children with simultaneous R/EV detection (MPP^RV^) and those with MPP (MPP^N^) alone was performed. Differences between these two groups and children with MPP infections in combination with other pathogens are shown in Table 2.

**Table 2:** 2024 patients’ clinical features compared between MPP^N^ vs. MPP^RV^

Constituents	MPP^*N*^ (*n* = 46)	MPP^RV^ (*n* = 25)	*p*-value
Age (y) – median (IQR)	10.55 (8.63)	7.11 (5.35) (24)	0.102^U^
Female Gender	**29 (63.0)**	**9 (37.5)**	**0.042** ^**C**^
Hospitalization	38 (82.6)	17 (68.0)	0.159^C^
Duration of Hospitalization – median (IQR)	5.00 (5.00)	7.00 (27.5)	0.330^U^
ICU Treatment	4 (8.7)	2 (8.0)	>0.999^FFH^
Respiratory Infection as Main Complaint at Admission:	34 (73.9)	14 (56.0)	0.123^C^
**Symptoms**			
General Respiratory Symptoms	41 (89.1)	23 (92.0)	>0.999^FFH^
Cough	39 (84.8)	20 (80.0)	0.742^FET^
Wheeze	7 (15.2)	7 (28.0)	0.223^FET^
General Gastrointestinal Symptoms	20 (43.5)	12 (48.0)	0.715^C^
Diarrhea	5 (10.9)	3 (12.0)	>0.999^FET^
Emesis	12 (26.1)	9 (36.0)	0.382^C^
Fever > 38,5 °C	**37 (80.4)**	**13 (52.0)**	**0.012** ^**C**^
**Pre-existing Conditions**	22 (47.8)	16 (66.7) (24)	0.133^C^
**Diagnostics**			
Laboratory examination	44 (95.7)	21 (84.0)	0.175^FET^
Chest X-Ray	32 (69.6)	13 (52.0)	0.142^C^
Treatment			
O2-Substitution	22 (47.8)	10 (40.0)	0.527^C^
Inhaled NaCl	28 (60.9)	12 (48.0)	0.497^FFH^
Inhaled Salbutamol	14 (30.4)	6 (24.0)	0.742^FFH^
Inhaled Adrenalin	1 (2.2)	0 (0.0)	>0.999^FFH^
Inhaled Atrovent	6 (13.0)	3 (12.0)	>0.999^FFH^
Inhaled Corticosteroids	3 (6.5)	3 (12.0)	0.786^FFH^
Systemic Corticosteroids	10 (21.7)	8 (32.0)	0.620^FFH^
Antibiotics	46 (100.0)	23 (92.0)	0.121^FET^

Values are presented as n (%) unless otherwise specified. Continuous variables are shown as mean (SD) or median (IQR). p-values were calculated using the χ² test (C), Fisher’s exact test (FET), Fisher–Freeman–Halton test (FFH), or Mann–Whitney U test (U), as appropriate.

Because not all data could be reconstructed retrospectively, analyses were performed using a pairwise exclusion approach

Children in MPP^RV^ tended to be younger (10.55 (8.63) vs. 7.11 (5.35) *p* = 0.102) and significantly more males were included as compared to MPP^N^ (15 [62.5%] vs. 17 [37.0%]; χ² [1] = 4.147, *p* = 0.042). Hospitalization rates, mean and median duration of stays showed no significant difference (7.0 days /IQR 28 days vs. 5.0 days/IQR 5 days). Pre-existing conditions were frequently observed in both groups, although children with MPP^RV^ tended to have a higher proportion of comorbidities (16 [66.7%] vs. 22 [47.8%]; χ² [1] = 2.256, *p* = 0.133).

Both groups presented with respiratory symptoms as primary complaint. General respiratory symptoms, cough, wheeze, and gastrointestinal symptoms occurred at similar rates across groups. Fever, however, was significantly more frequently present in patients with MPP^N^ (37 [80.4%] vs. 13 [52.0%], χ² [1] = 6.287, *p* = 0.012).

Treatment did not differ significantly between groups regarding oxygen supplementation, inhaled medication, systemic corticosteroid administration, or total antibiotic use (Table 2).

### Marker of immune response in patients with MPP^N^ and MPP^RV^

Laboratory parameters were compared between patients with MPP^N^ and those with MPP^RV^.

**Fig. 3:**
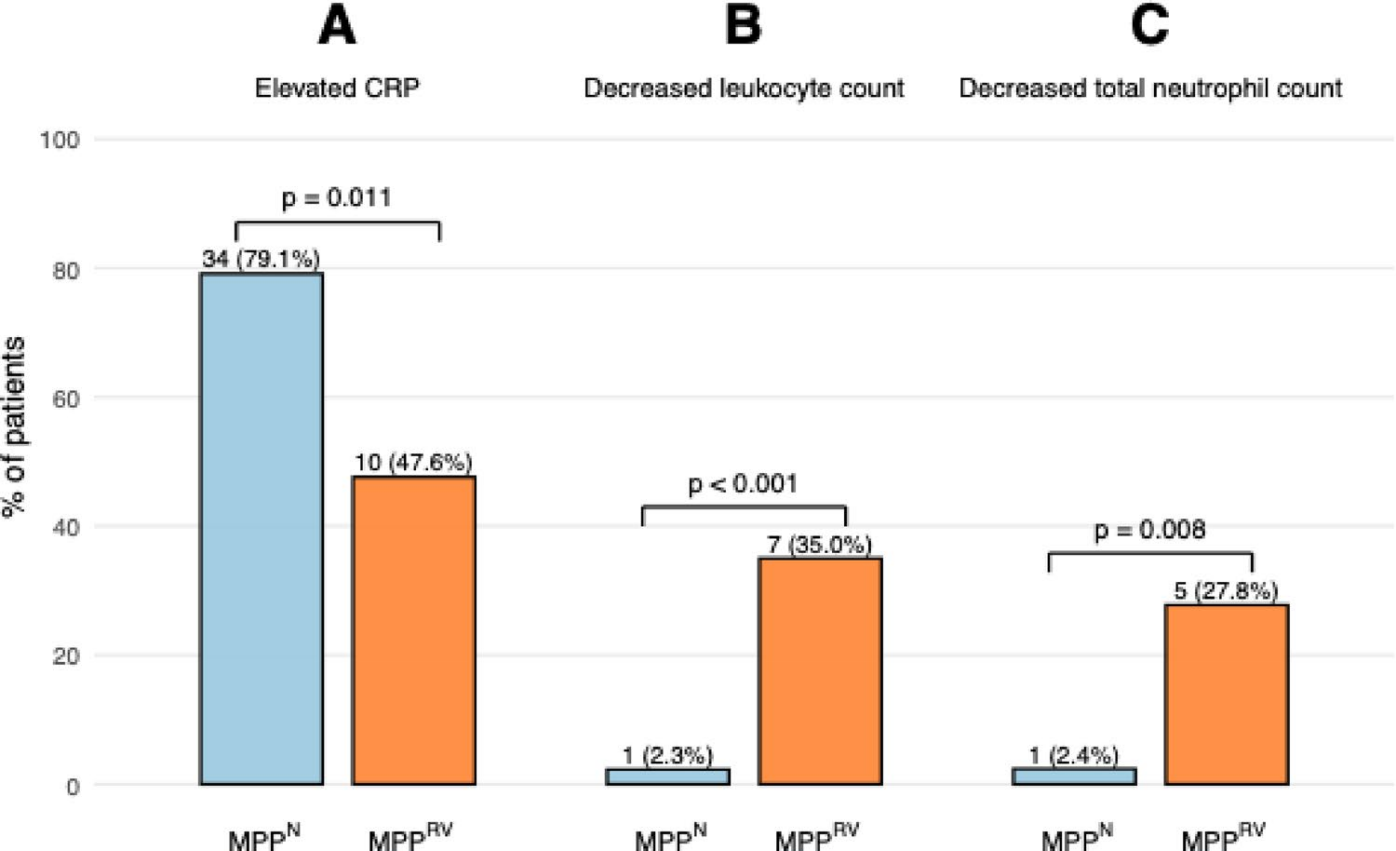
Laboratory markers in children with MP alone vs. MP + R/EV co-infection. Proportion of patients with **A** elevated CrP values, **B** decreased total leukocyte counts or **C** decreased total neutrophil counts Group differences were tested using χ² test or Fisher’s exact test (FET) Significant p-values are shown above the bars

Analysis of inflammatory markers revealed that elevated CrP levels were observed more frequently in patients with MPP^N^ compared to MPP^RV^ (34/43 [79.1%] vs. 10/21 [47.6%]; χ² [1] = 6.496, *p* = 0.011). In contrast, reduced total leucocyte count occurred significantly more often in MPP^RV^ (7/20 [35.0%]) than in MPP^N^ (1/43 [2.3%]; FET, *p* < 0.001). Similarly, decreased neutrophil counts were observed more frequently in MPP^RV^ (5/18 [27.8%]) than in MPP^N^ (1/41 [2.4%]; FET, *p* = 0.008) **(**Fig. 3**).**

Mean hemoglobin levels differed significantly between the two groups (t(27.9) = 2.36, *p* = 0.025), with lower values in the MPP^RV^ group (10.78 (2.53) g/dL) compared to MPP^N^ (12.25 (1.76) g/dL) (Table E5).

## Discussion

This study describes a marked increase in pediatric PCR-confirmed MPP detections during 2023/24 at a tertiary children’s hospital. Compared with the sporadic detections observed between 2017 and 2022, the 2023/24 period was characterized by substantially higher case numbers and frequent viral co-detections, particularly RV/EV. Because the historical comparison group was small, these data should primarily be interpreted as descriptive evidence of local resurgence rather than as proof of statistically robust differences between periods. Despite this increase, the epidemiological and clinical characteristics of the patient cohort were similar to those observed in prior years, and co-detections remained consistently common. Within the 2023/24 cohort, children with MPP plus RV/EV co-detection differed from those with MPP mono-detection in sex distribution, fever frequency, and selected inflammatory parameters. These findings suggest that RV/EV co-detection may be associated with a distinct clinical and laboratory pattern. However, the retrospective design does not allow conclusions regarding causality, disease-modifying effects, or the temporal sequence of infection.

The marked decline in MPP detection rates during 2020–2022, followed by the observed increase beginning in 2023 and the further rise in 2024, has been independently documented in many regions of the world [27–29]. This phenomenon may be attributable to the lifting of non-pharmaceutical interventions (NPIs), including the use of face masks, physical distancing, and the general reduction in contact with individuals outside one’s own household, implemented through closures of community settings such as daycare centers and schools, as well as lockdowns in general. The resurgence of various respiratory pathogens following the relaxation of NPIs has been demonstrated in several studies [30–32].

One possible explanation is the concept of immunity debt, which describes how temporarily reduced exposure to common pathogens, along with a decline in vaccinations, leads to a reduction in non-specific immunity and ultimately to increased susceptibility to respiratory infectious disease upon renewed exposure to infectious agents [33, 34]. However, the key difference between MPP and other respiratory pathogens lies in the fact that MPP detections remained consistently low throughout the COVID-19 pandemic [18, 19, 28], whereas many pathogens returned relatively quickly during the first phases of NPI relaxation or were entirely unaffected by the measures [21].

The reason for the delayed re-emergence of MPP infections after the COVID-19 pandemic, especially in comparison to other respiratory pathogens, remains incompletely understood. Additional considerations include the cyclical pattern of MPP infections. The last notable increase in MPP cases in Germany occurred in 2017/2018 [16, 19]. Thus, the resurgence beginning in 2023 and escalating in 2024 may fall within the typical MPP cycle. Combined with the presumed increased susceptibility of the population due to waning herd immunity, this may underline the pronounced peak observed in 2024.

Co-detection with R/EV was associated with alterations in the patient characteristics. MPP^RV^ patients were younger and more often male This reflects the characteristic epidemiological findings of R/EV infections. While MPP is considered to affect mainly older children [12, 35], rhinovirus – especially severe cases – are seen in younger patients [36].

The co-occurrence of MPP with other respiratory pathogens has been described in several studies. In some of these studies, rhinovirus was among the most frequently detected pathogens, identified alongside MPP in up to 30% of coinfection cases [37–39]. Similar findings were observed in our G2 (32.9% of patients tested positive for R/EV). One biologically plausible mechanism involves intercellular adhesion molecule 1 (ICAM-1), which is induced during MPP interaction with respiratory epithelium and is also used as a receptor by many rhinovirus serotypes. MPP induces increased expression of ICAM-1 during interaction with the respiratory epithelium [5]. Viral pathogens can utilize surface receptors such as ICAM-1 to initiate infection. As early as 1989, it was demonstrated that a large number of rhinovirus serotypes use ICAM-1 to mediate viral internalization [40]. Thus, an initial MPP infection may facilitate subsequent infection of the respiratory epithelium with rhinovirus. However, our study cannot determine whether MPP preceded RV/EV infection, whether RV/EV preceded MPP infection, or whether both pathogens were detected during overlapping but independent infections. Because antibody titers, pathogen loads, and serial samples were not available, this hypothesis remains speculative and requires prospective investigation.

Both RV and MPP have previously been associated with wheezing, airway inflammation, and asthma exacerbations in children. Rhinovirus (RV) is recognized as the most common viral trigger of wheezing in children, and early-life RV-induced wheezing episodes are strong predictors of subsequent asthma development [41, 42]. RV infection contributes to airway remodeling through epithelial damage, smooth muscle proliferation, and fibroblast activation, leading to persistent airway narrowing and reduced responsiveness to bronchodilators [43]. In addition, RV alters immune signaling, particularly interferon responses, which are often impaired in individuals predisposed to asthma. Children with asthma exhibit epithelial traits that facilitate RV entry, reinforcing a cycle of infection and airway injury [41]. *Mycoplasma pneumoniae* (MPP) is a bacterial pathogen implicated in both the initiation and exacerbation of asthma. MPP infection stimulates Th2-type immune responses, including the release of IL-4, IL-5, and IL-13, which enhance airway hyperresponsiveness and allergic pathways [44]. Chronic or recurrent MPP infection sustains low-grade inflammation, maintaining the airways in a primed state for exaggerated responses to future triggers [45]. Evidence further suggests that MPP infection can directly induce asthma onset in susceptible individuals, underscoring its role as both a trigger and perpetuator of airway disease [9]. In this context, the high frequency of RV/EV co-detection in children with MPP is clinically relevant, particularly among children with pre-existing respiratory disease. However, our study did not assess long-term outcomes, recurrent wheezing, lung function, or later asthma development. Therefore, any link between MPP–RV/EV co-detection and chronic airway disease should be regarded as hypothesis-generating. Prospective studies with follow-up are needed to determine whether children with combined detections are at increased risk for persistent respiratory morbidity.

Potential synergistic effects of RV and MPP coinfection appear to exert synergistic effects on airway pathology. Such coinfections intensify epithelial injury and impair barrier function, rendering the airway more vulnerable to allergens and pollutants. The combined viral and bacterial immune activation results in excessive cytokine release (IL-4, IL-5, IL-13), fueling eosinophilic inflammation—a hallmark of asthma [46]. Coinfection may also skew immune development in children, promoting allergic sensitization and long-term airway hyperreactivity [47, 48]. Importantly, children with RV-induced wheezing who also harbor MPP are at higher risk of persistent asthma compared to those with viral infection alone [46]. The high prevalence of RV/EV and MPP coinfections observed in our clinic raises important considerations. With RV driving acute wheezing and airway remodeling, and MPP sustaining inflammation and immune dysregulation, a “double-hit” mechanism must be accounted for.

This study has several limitations. First, the retrospective single-center design limits causal inference and generalizability. Second, the historical comparison group was very small, with only ten cases between 2017 and 2022. Therefore, comparisons between the historical period and the 2023/24 resurgence should be interpreted primarily as descriptive background rather than as robust statistical evidence of clinical differences.

Third, the subgroup analysis comparing MPP mono-detection with MPP plus RV/EV co-detection was exploratory. Sample sizes were modest, multiple comparisons were performed, and adjustment for potential confounders such as age, sex, and comorbidities was limited by sample size. The observed associations therefore require confirmation in larger cohorts.

Fourth, PCR positivity indicates pathogen detection but does not necessarily prove active infection. This is particularly relevant for MPP, as asymptomatic carriage has been described. Nevertheless, most included children presented with respiratory symptoms, supporting clinical relevance in many cases. Still, the distinction between detection, co-detection, and true coinfection cannot be fully resolved retrospectively.

Fifth, no antibody titers, pathogen loads, or serial respiratory samples were available. As a result, the temporal sequence of MPP and RV/EV detection could not be determined. Mechanistic interpretations regarding pathogen interaction or immune modulation must therefore remain speculative.

Finally, missing data were handled by pairwise exclusion, leading to variable denominators across analyses, particularly for laboratory parameters. A major strength of this study is the unique temporal positioning during the post-pandemic resurgence of respiratory pathogens, allowing for a detailed characterization of a phenomenon that has so far only been described epidemiologically but not clinically. The consistent diagnostic approach, the age-adjusted analysis of inflammatory markers, and the unusually high incidence of R/EV coinfections provide a clinically meaningful dataset that offers new insights into pathogen interactions during this period of rapid epidemiological change.

## Conclusion

The 2023/24 period was characterized by a marked increase in pediatric PCR-confirmed Mycoplasma pneumoniae detections at our center. RV/EV was frequently co-detected, and children with MPP plus RV/EV showed differences in fever frequency and inflammatory laboratory parameters compared with children with MPP alone. These findings suggest that RV/EV co-detection may be associated with a distinct clinical phenotype during the MPP resurgence. However, because this was a retrospective single-center study with modest subgroup sizes and no serial microbiological sampling, the results should be interpreted as associative and hypothesis-generating. Larger prospective studies are needed to clarify the clinical relevance, temporal sequence, and potential biological interactions of MPP and RV/EV in children.

## Supplementary Information

Below is the link to the electronic supplementary material.

Supplementary Material 1

## Data Availability

All data supporting the findings of this study are available within the paper and its Supplementary Information.

## Abbreviations

CrP C: reactive Protein
FET: Fisher’s exact test
G1: Group 1; Pre-resurgence (2017-2022)
G2: Group 2; Resurgence (2023-2024)
ICAM-1: intercellular adhesion molecule 1
IQR: Inter quartile range
MPP: Mycoplasma pneumoniae
MPP^N^: Mycoplasma pneumoniae without Rhinovirus/enterovirus
MPP^RV^: Mycoplasma pneumoniae plus Rhinovirus/enterovirus
NPI: Non-pharmaceutical interventions
R/EV: Rhinovirus/enterovirus
RSV: Respiratory syncytial virus
S. pneumoniae: Streptococcus pneumoniae
SD: Standard deviation
U-test: Mann–Whitney U test
