# Supplementary material for: Post-pandemic resurgence of Mycoplasma pneumoniae and its emerging link with Rhinovirus in children
Source: Infection. 2026 May 27;54(4):2041–51. doi: 10.1007/s15010-026-02833-8 (PMC13476293; doi:10.1007/s15010-026-02833-8)
Supplement: Supplementary file 1 [file 15010_2026_2833_MOESM1_ESM.docx]

**Supplemental Material**

**Figure S1**

Excluded (n = 142):

- Incomplete retrospective data (n = 94)
- Detection by non-PCR methods (n = 32)
- Clinical diagnosis only (n = 8)
- Age > 18 years (n = 4)
- Duplication of cases (n = 4)

***Group 2***

**Post-COVID cases (2024),**

n = 87*

**Children with suspected MPP infection, 2017–2024**

n = 239

**Confirmed MPP infection by PCR, 2017–2024**

n = 97

***Group 1***

***Pre-peak cases (2017–2022),***

n = 10

**FIG. S1. Flowchart of patient selection and stratification into pre- and post-peak MPP groups**

Reinfection was defined as a renewed positive MPP detection with an interval of > 1 month from the previous pathogen detection.

*One patient had two independent PCR-confirmed MPP episodes (>1 month apart), therefore counted as separate cases.

**Table E1**

| TABLE E1. Age and gender-dependent reference range of laboratory parameters | | | | |
| --- | --- | --- | --- | --- |
| Laboratory parameters | **Age** | **Gender** | **Reference range** | **Unit** |
| *C-reactive protein* | 1 - 30 Days | b* | no reference range | mg/dl |
|  | 1 - 3 Months | b | ≤ 1.6 | mg/dl |
|  | 3 Months - 4 Years | m* | ≤ 1.1 | mg/dl |
|  |  | f* | ≤ 0.8 | mg/dl |
|  | 4 - 18 Years | m | ≤ 0.8 | mg/dl |
|  | 4 - 11 Years | f | ≤ 1.0 | mg/dl |
|  | 11 - 18 Years | f | ≤ 0.8 | mg/dl |
| Total Leucocyte Count | 1 - 16 Days | m | 8.04 - 15.40 | G/l |
|  |  | f | 8.16 - 14.56 | G/l |
|  | 17 - 32 Days | m | 7.80 - 15.91 | G/l |
|  |  | f | 8.36 - 14.42 | G/l |
|  | 33 - 62 Days | m | 8.14 - 14.99 | G/l |
|  |  | f | 7.05 - 14.68 | G/l |
|  | 63 - 182 Days | m | 6.51 - 13.32 | G/l |
|  |  | f | 6.00 - 13.25 | G/l |
|  | 6 Months - 9 Years | b | 3.50 - 14.00 | G/l |
|  | 9 - 18 Years | b | 4.10 - 12.00 | G/l |
| Hemoglobin | 1 - 16 Days | m  f | 13.9 - 19.1  13.4 - 20.0 | g/dl  g/dl |
|  | 17 - 32 Days | m  f | 10.0 - 15.3  10.8 - 14.6 | g/dl  g/dl |
|  | 33 - 62 Days | m  f | 8.9 - 12.7  9.2 - 11.4 | g/dl  g/dl |
|  | 63 - 182 Days | m  f | 9.6 - 12.4  9.9 - 12.4 | g/dl  g/dl |
|  | 6 Months - 8 Years | b | 10.7 - 13.4 | g/dl |
|  | 8 - 12 Years | b | 11.8 - 14.8 | g/dl |
|  | 12 Years - 18 Years | m  f | 12.6 - 17.0  11.2 - 15.8 | g/dl  g/dl |
| Total thrombocyte count | 1 - 16 Days | m  f | 218 - 419  144 - 449 | G/l  G/l |
|  | 17 - 32 Days | m  f | 248 - 586  279 - 571 | G/l  G/l |
|  | 33 - 62 Days | m  f | 229 - 562  331 - 597 | G/l  G/l |
|  | 63 - 182 Days | m  f | 244 - 529  247 - 580 | G/l  G/l |
|  | 6 Months - 11 Years | b | 195 - 464 | G/l |
|  | 11 - 18 Years | b | 215 - 594 | G/l |
| Total Neutrophil count | 1 - 16 Days | m  f | 1.60 - 6.06  1.73 - 6.75 | G/l  G/l |
|  | 17 - 32 Days | m  f | 1.18 - 5.45  1.23 - 4.80 | G/l  G/l |
|  | 33 - 62 Days | m  f | 0.83 - 4.23  1.00 - 4.68 | G/l  G/l |
|  | 63 - 182 Days | m  f | 0.97 - 5.45  1.04 - 7.2 | G/l  G/l |
|  | 6 Months - 15 Years | b | 1.57 - 6.70 | G/l |
|  | 15 - 18 Years | b | 2.01 - 9.55 | G/l |
| Total Lymphocyte count | 1 - 16 Days | m  f | 2.07 - 7.53  1.75 - 8.00 | G/l  G/l |
|  | 17 - 32 Days | m  f | 2.11 - 8.38  2.42 - 8.20 | G/l  G/l |
|  | 33 - 62 Days | m  f | 2.47 - 7.95  2.29 - 9.14 | G/l  G/l |
|  | 63 - 182 Days | m  f | 2.45 - 8.89  2.14-8.99 | G/l  G/l |
|  | 6 Months - 6 Years | b | 1.83 - 7.85 | G/l |
|  | 6 - 13 Years | b | 1.32 - 4.07 | G/l |
|  | 13 - 18 Years | b | 1.22 - 3.55 | G/l |
| Total Basophil Count | 1 - 16 Days | m  f | 0.02 - 0.11  0.02 - 0.07 | G/l  G/l |
|  | 17 - 32 Days | m  f | 0.01 - 0.07  0.01 - 0.06 | G/l  G/l |
|  | 33 - 62 Days | m  f | 0.01 - 0.06  0.01 - 0.05 | G/l  G/l |
|  | 63 - 182 Days | m  f | 0.01-0.06  0.01-0.07 | G/l  G/l |
|  | 6 Months - 18 Years | b | 0.01 - 0.10 | G/l |
| Total Eosinophil Count | 1 - 16 Days | m  f | 0.12 - 0.66  0.09 - 0.64 | G/l  G/l |
|  | 17 - 32 Days | m  f | 0.08 - 0.80  0.06 - 0.75 | G/l  G/l |
|  | 33 - 62 Days | m  f | 0.05 - 0.57  0.04 - 0.63 | G/l  G/l |
|  | 63 - 182 Days | m  f | 0.03 - 0.61  0.02-0.74 | G/l  G/l |
|  | 6 Months - 14 Years | b | 0.05 - 0.69 | G/l |
|  | 14 - 18 Years | b | 0.03 - 0.59 | G/l |

*b (male and female), f (female), m (male)

**Table E2**

| **TABLE E2. Distribution of Pre-Existing Condition Categories (Multiple Responses)** | | | | | | |
| --- | --- | --- | --- | --- | --- | --- |
|  | **Group 1**  **2017 – 2022**  **(n = 10)** | | | **Group 2**  **2023 – 2024**  **(n = 86)*** | | |
| **Categories of Pre-existing Conditions** | **Amount (n)** | **Percent**  **(%)** | **Percent of Cases (%)** | **Amount (n)** | **Percent**  **(%)** | **Percent of Cases (%)** |
| Neurological PC | / | / | / | 14 | 18.9 | 28.6 |
| Hematooncological PC | 2 | 18.2 | 25.0 | 11 | 14.9 | 22.4 |
| Respiratory PC | 3 | 27.3 | 37.5 | 12 | 16.2 | 24.5 |
| Cardiological PC | 1 | 9.1 | 12.5 | 9 | 12.2 | 18.4 |
| Immunological PC | 2 | 18.2 | 25.0 | 7 | 9.5 | 14.3 |
| Neonatal PC | 1 | 5.3 | 7.7 | 5 | 6.8 | 10.2 |
| Genetical PC | 2 | 18.2 | 25.0 | 5 | 6.8 | 10.2 |
| Gastroenterological PC | / | / | / | 3 | 4.1 | 6.1 |
| Endocrinological PC | / | / | / | 2 | 2.7 | 4.1 |
| Nephrological PC | / | / | / | 2 | 2.7 | 4.1 |
| Dermatological PC | / | / | / | 1 | 1.4 | 2.0 |
| Metabolical PC | 1 | 9.1 | 12.5 | 1 | 1.4 | 2.0 |
| Ophthalmological PC | / | / | / | 1 | 1.4 | 2.0 |
| Psychiatric PC | / | / | / | 1 | 1.5 | 2.0 |
| **In Total** | 19 | 100.0 | 146.2 | 67 | 100.0 | 148.9 |
| Multiple responses per patient were possible; therefore, percentages may exceed 100%. The table presents the absolute number of conditions (n), the proportion of all documented pre-existing conditions within each cohort (%), and the proportion relative to the total number of cases in the respective cohort (% of cases).  *PID_078 was accounted for once. | | | | | | |

**Table E3**

| **TABLE E3.** Laboratory findings in the Group 1 (2017 – 2022) and Group 2 (2023 – 2024) | | | |
| --- | --- | --- | --- |
| **Laboratory Parameter** | **Group1**  **(G1, 2017 – 2022)**  **n = 10** | **Group 2**  **(G2, 2023 – 2024)**  **n = 87** | **p-value** |
| Elevated CRP | 8 (80.0) | 51 (67.1) (76) | 0.495^FET^ |
| CRP (mg/L) – median (IQR) | 3.45 (6.60) | 2.30 (4.75) | 0.284^U^ |
| Elevated leukocyte count | 0 (0.0) (9) | 17 (22.7) (75) | 0.193^FET^ |
| Decreased leukocyte count | 2 (22.2) (9) | 9 (12.0) (75) | 0.335^FET^ |
| Leucocytes (G/L) – median (IQR) | 7.82 (9.27) | 8.75 (6.19) | 0.315 ^U^ |
| Elevated hemoglobin | 2 (22.2) (9) | 6 (8.0) (75) | 0.203^FET^ |
| Decreased hemoglobin | 3 (33.3) (9) | 20 (26.7) (75) | 0.700^FET^ |
| Hemoglobin (g/dL) – median (IQR) | 11.90 (5.00) | 11.90 (2.30) | 0.492 ^U^ |
| Elevated platelets | 1 (11.1) (9) | 4 (5.3) (75) | 0.441^FET^ |
| Decreased platelets | 1 (11.1) (9) | 19 (25.3) (75) | 0.679^FET^ |
| Platelets (G/L) – mean (SD) | 283.89 (103.21) | 283.93 (134.97) | 0.428^t^ |
| Elevated total neutrophil count | 3 (33.3) (9) | 23 (32.4) (71) | >0.999^FET^ |
| Decreased total neutrophil count | 2 (22.2) (9) | 7 (9.9) (71) | 0.^266FET^ |
| Neutrophils (G/L) – median (IQR) | 2.96 (7.22) | 5.37 (5.29) | 0.193^U^ |
| Elevated total lymphocyte count | 0 (0.0) (9) | 1 (1.4) (71) | >0.999^FET^ |
| Decreased total lymphocyte count | 3 (33.3) (9) | 25 (35.2) (71) | >0.999^FET^ |
| Lymphocytes (G/L) – median (IQR) | 1.79 (2.94) | 1.75 (1.49) | 0.698^U^ |
| Elevated total eosinophil count | 0 (0.0) (9) | 3 (4.2) (70) | >0.999^FET^ |
| Decreased total eosinophil count | 4 (44.4) (9) | 28 (39.4) (70) | >0.999 ^FET^ |
| Eosinophils (G/L) – median (IQR) | 0.05 (0.15) | 0.09 (0.24) | 0.295^U^ |
| Elevated total basophil count | 0 (0.0) (8) | 2 (3.1) (65) | >0.999^FET^ |
| Basophils (G/L) – median (IQR) | 0.035 (0.043) | 0.030 (0.020) | 0.699^U^ |
| Values are given as *n (%)* (n tested) unless otherwise specified. Continuous data are expressed as mean (SD) or median (IQR). *p*-values were calculated using Fisher’s exact (FET) and Mann–Whitney U (U), or Student’s *t* (t) tests, as appropriate. Because not all patients underwent all laboratory analyses, a pairwise exclusion approach was applied. One MPP^N^ patient was excluded from the laboratory analysis for not meeting the inclusion criteria. | | | |

| **TABLE E4. Distribution of co-infections in Group 1 (2017 – 2022) and Group 2 (2023 – 2024)** | | | |
| --- | --- | --- | --- |
| **Detected Pathogens** | **Group 1**  **(G1, 2017-2022)**  **n = 10** | **Group 2**  **(G2 2023-2024)**  **n = 87** | **p-value** |
|  |  |  |  |
| Rhinovirus | 0 (0.0) | 27 (31.0) | 0.057 |
| Influenza A | 1 (10.0) | 0 (0.0) | 0.103 |
| *H. influenzae* | 1 (10.0) | 2 (2.3) | 0.281 |
| Coronavirus (not SARS-CoV-2) | 1 (10.0) | 0 (0.0) | 0.103 |
| *S. pneumoniae* | 1 (10.0) | 0 (0.0) | 0.103 |
| SARS-CoV-2 | 0 (0.0) | 3 (2.7) | >0.999 |
| Adenovirus | 0 (0.0) | 9 (10.3) | 0.591 |
| Respiratory Syncytial Virus | 0 (0.0) | 3 (3.4) | >0.999 |
| Parainfluenca virus | 0 (0.0) | 4 (4.6) | >0.999 |
| *Chlamydia pneumoniae* | 0 (0.0) | 1 (1.1) | >0.999 |
| *Bordetella pertussis/parapetussis* | 1 (10.0) | 0 (0.0) | 0.103 |
| *Moraxella catarrhalis* | 1 (10.0) | 0 (0.0) | 0.103 |
| Values are given as *n (%)* (n tested) unless otherwise specified.  *p*-values were calculated using Fisher’s exact (FET). | | | |

**Table E4**

| **TABLE E5. Laboratory findings compared between MPP alone and MPP + R/EV** | | | |
| --- | --- | --- | --- |
| **Laboratory Parameter** | **MPP^N^**  **(n = 46)** | **MPP^RV^**  **(n = 25)** | **p-value** |
| Elevated CRP | **34 (79.1) (43)** | **10 (47.6) (21)** | **0.011^C^** |
| CRP (mg/L) – median (IQR) | 2.70 (3.50) | 0.80 (4.95) | 0.232^U^ |
| Elevated leukocyte count | 11 (25.6) (43) | 3 (15.0) (20) | 0.518^FET^ |
| Decreased leukocyte count | **1 (2.3) (43)** | **7 (35.0) (20)** | **<0.001^FET^** |
| Leucocytes (G/L) – median (IQR) | 8.88 (6.12) | 7.14 (9.29) | 0.056^U^ |
| Elevated hemoglobin | 2 (4.7) (43) | 3 (15.0) (20) | 0.315^FET^ |
| Decreased hemoglobin | 9 (20.9) (43) | 8 (40.0) (20) | 0.112^C^ |
| Hemoglobin (g/dL) – mean (SD) | **12.25 (1.76)** | **10.78 (2.53)** | **0.025^T^** |
| Elevated platelets | 1 (2.3) (43) | 1 (5.0) (20) | 0.538^FET^ |
| Decreased platelets | **9 (20.9) (43)** | **9 (45.0) (20)** | **0.049^C^** |
| Platelets (G/L) – mean (SD) | 291.44 (112.24) | 221.2 (164.50) | 0.095^T^ |
| Elevated total neutrophil count | 14 (34.1) (41) | 3 (16.7) (18) | 0.172^C^ |
| Decreased total neutrophil count | **1 (2.4) (41)** | **5 (27.8) (18)** | **0.008^FET^** |
| Neutrophils (G/L) – median (IQR) | **5.37 (5.56)** | **3.68 (5.95)** | **0.031^U^** |
| Elevated total lymphocyte count | 1 (2.4) (41) | 0 (0.0) (18) | >0.999^FET^ |
| Decreased total lymphocyte count | 12 (29.3) (41) | 8 (44.4) (18) | 0.257^C^ |
| Lymphocytes (G/L) – median (IQR) | 1.75 (1.21) | 1.78 (1.90) | 0.401^U^ |
| Elevated total eosinophil count | 2 (4.9) (41) | 1 (5.6) (18) | >0.999^FET^ |
| Decreased total eosinophil count | 17 (41.5) (41) | 5 (27.8) (18) | 0.317^C^ |
| Eosinophils (G/L) – median (IQR) | 0.08 (0.24) | 0.15 (0.37) | 0.434^U^ |
| Elevated total basophil count | 2 (5.0) (40) | 0 (0.0.) (13) | >0.999^FET^ |
| Basophils (G/L) – median (IQR) | 0.030 (0.020) | 0.030 (0.050) | 0.925^U^ |
| Values are given as n (%) (n tested) unless otherwise specified. Continuous data are expressed as mean (SD) or median (IQR). p-values were calculated using χ² (C), Fisher’s exact (FET), Mann–Whitney U (U), or Welch t (T) tests, as appropriate. Because not all patients underwent all laboratory analyses, a listwise exclusion approach was applied. One MPP^N^ patient was excluded from the laboratory analysis for not meeting the inclusion criteria. | | | |

**Table E5**
